# Effects of beta-glucan in a mice model of vaginitis by candida albicans

**DOI:** 10.1186/1939-4551-8-S1-A227

**Published:** 2015-04-08

**Authors:** Valéria Soraya De Farias Sales, Aleida Maria Da Silva Lima, Karina Mendes Melchuna, Luanda Bárbara Ferreira Canário Souza, Mariana Araújo Costa, Guilherme Maranhão Chaves, Sarah Dantas Viana Medeiros

**Affiliations:** 1 Federal University of Rio Grande Do Norte, Brazil

## Background

Vulvovaginal candidiasis is an inflammatory disease, on vaginal tissue, caused mainly by pathogenic yeasts of *Candida albicans.* The objective of this study was to evaluate the immunomodulatory activity of beta-glucan in mice with vulvovaginal candidiasis under the influence of estrogen.

## Methods

Fifty four Balb/C mice of 7 to 10 weeks old under influence of estrogen were inoculated with 5x10^4^ stationary-phase blastoconidia of *C. albicans*, intravaginally. The mice were divided in three groups, treated with glucan vaginally (5mg/mL) and intraperitoneally (1mg/mL), and the control group that received saline, intraperitoneally. Vaginal lavage was obtained on days 2, 5, 8 e 10 after inoculation with *C. albicans* for the count of CFU by pour-plate method. Moreover, in the days 6, 9 and 11 after inoculation, three mice from each group were sacrificed and the vaginas were removed for histophatological analysis. The slides were stained by hematoxylin-eosin to evaluate the infiltrate of neutrophils and periodic acid-Schiff (PAS) for analyze of fungal burden.

## Results

The mice treated with intraperitoneal and vaginal glucan showed smaller number of the CFU of *C. albicans* in the vaginal fluid, compared with control mice. However, just intraperitoneal group showed decrease of CFU statistically significant of 1.98 folds (p<0,01), in the 8 day that was confirmed with histopathological analysis. The groups treated with glucan showed greater infiltration of neutrophilis compared with control group, but only vaginal group showed increase of neutrophilis statistically significant of 3,3 fold (p<0,01), in the 9 day, compared to control group.

## Conclusions

The data suggest that glucan may have an important activity in protection against vulvovaginal candidiasis associated to *C. albicans*.

